# Metabolism-Regulating Microspheres: Design Principles, Therapeutic Applications Across Multisystem Diseases, and Future Perspectives

**DOI:** 10.34133/research.1106

**Published:** 2026-01-27

**Authors:** Shengwen Cheng, Lijun Yang, Mingfei Dong, Xiaohong Luo, Xinle Zhang, Yu Zhai, Yi He, Yuqiao Wang, Xiaole Peng, Xiaoxiong Li, Yichi Zhang, Chen Zhao, João F. Mano, Wei Huang, Yiting Lei

**Affiliations:** ^1^ Department of Orthopedic Surgery, The First Affiliated Hospital of Chongqing Medical University, Chongqing 400016, China.; ^2^ Chongqing Municipal Health Commission Key Laboratory of Musculoskeletal Regeneration and Translational Medicine, Chongqing 400016, China.; ^3^ Orthopedic Research Laboratory of Chongqing Medical University, Chongqing 400016, China.; ^4^ Department of Obstetrics and Gynecology, The First Affiliated Hospital of Chongqing Medical University, Chongqing 400016, China.; ^5^ Clinical Medical College & Affiliated Hospital of Chengdu University, Chengdu University, Chengdu 610081, China.; ^6^ Ophthalmology Medical Center, The First Affiliated Hospital of Chongqing Medical University, Chongqing Key Laboratory for the Prevention and Treatment of Major Blinding Eye Diseases, Chongqing Branch (Municipality Division) of National Clinical Research Centre for Ocular Diseases, Chongqing 400016, China.; ^7^ Department of Rehabilitation Medicine, Key Laboratory of Physical Medicine and Precision Rehabilitation of Chongqing Municipal Health Commission, The First Affiliated Hospital of Chongqing Medical University, No.1 Youyi Road, Yuzhong District, Chongqing 400016, China.; ^8^ Department of Chemistry, CICECO - Aveiro Institute of Materials, University of Aveiro, Campus Universitário de Santiago, Aveiro 3810-193, Portugal.; ^9^ Department of Biomedical Engineering, The Chinese University of Hong Kong, NT, Hong Kong SAR 999077, China.

## Abstract

Metabolism-regulating microspheres have evolved from conventional drug carriers into active platforms capable of spatiotemporally reprogramming pathological metabolic networks. Chronic diseases are increasingly understood to be driven by metabolic dysregulation, highlighting the need for therapeutic strategies that enable localized and precise metabolic intervention. This review systematically outlines the core design principles of these microspheres, emphasizing the synergistic integration of engineered chemical properties, such as ionic signaling, metabolite delivery, and pathway modulator release, with tailored physical characteristics, including stiffness, porosity, and size of the microspheres. Together, these features construct “metabolic instruction systems” that correct dysregulated pathways at the tissue level. Their versatile applications include orthopedic diseases, such as osteoporosis, osteoarthritis, and bone defects; ophthalmic conditions, including glaucoma and diabetic retinopathy; and gynecological disorders, such as premature ovarian insufficiency, ovarian cancer, and endometriosis. These systems target key metabolic abnormalities, such as glycolytic dysregulation, mitochondrial dysfunction, and oxidative stress, which are recognized as central drivers of disease pathogenesis across multiple organ systems. Despite considerable progress, clinical translation remains limited by tissue-specific delivery barriers, interindividual metabolic heterogeneity, and long-term safety concerns within dynamic metabolic networks. Emerging strategies, such as personalized formulations, artificial-intelligence-driven designs, and organ-on-a-chip validation platforms, are being developed to address these challenges. With ongoing interdisciplinary innovation, metabolism-regulating microspheres hold great promise as precise therapeutic modalities for a spectrum of chronic diseases rooted in metabolic imbalance, offering targeted and sustained metabolic correction.

## Introduction

The pathogenesis of chronic diseases has undergone fundamental reconceptualization, with metabolic dysregulation transitioning from a correlative phenomenon to a recognized causal driver. Rather than being a secondary consequence, aberrant metabolic activity functions as a pivotal regulator that can initiate and sustain complex pathological cascades. This regulatory influence stems from the governance of integrated cellular metabolic networks that coordinate energy production, biosynthetic output, and signaling events to direct cellular fate and maintain homeostasis [1]. The profound interconnectivity of these pathways indicates that perturbations within one metabolic network frequently cascade into systemic imbalances, establishing a common pathogenic nexus across different organ systems. Disruption of this delicate homeostatic equilibrium creates a permissive environment for disease initiation and progression in various tissues. For instance, in osteoarthritis (OA), a shift toward glycolytic metabolism and mitochondrial dysfunction in chondrocytes drives procatabolic and inflammatory responses that destroy articular cartilage; a glycolytic switch drives uncontrolled proliferation in tumor cells; and mitochondrial failure triggers neuronal demise in neurodegenerative disorders [2,3]. This paradigm shift underscores the therapeutic potential of rectifying core metabolic imbalances for disease modification in a broad range of conditions. Notably, metabolic stress such as amino acid starvation can also disrupt fundamental cellular rhythms, as demonstrated in fungi where the general control nonderepressible 2 pathway maintains the circadian clock via histone acetylation at the frq promoter [4]. This highlights that metabolic perturbations not only drive pathological cascades but also impair endogenous regulatory cycles essential for homeostasis. Therefore, interventions capable of locally restoring metabolic equilibrium may also help preserve or restore such biological rhythms in diseased tissues.

The growing appreciation for the pathogenic role of metabolism has positioned metabolic reconfiguration—the targeted remodeling of cellular energy and biosynthetic pathways without disrupting systemic homeostasis—as a frontier in therapeutic science. However, conventional pharmacological approaches to metabolic intervention, which primarily rely on the systemic administration of enzyme inhibitors or metabolic precursors, have substantial limitations. This fundamental lack of tissue specificity often leads to off-target effects, compromised therapeutic efficacy and dose-limiting toxicity. These shortcomings manifest clinically as narrow therapeutic windows and compensatory metabolic adaptations that undermine sustainability [5]. Therefore, the critical challenge lies in developing innovative strategies capable of correcting tissue-specific metabolic dysregulation while preserving global metabolic equilibrium, which requires advanced delivery platforms that enable spatially confined and temporally controlled metabolic interventions.

Engineered microspheres have emerged as transformative platforms for localized metabolic regulation, representing a paradigm shift from conventional drug delivery systems [6]. Beyond their traditional role as passive, controlled-release depots, contemporary microspheres function as dynamic metabolic interfaces that engage and reprogram pathological processes. This transition from passive carriage to active metabolic modulation is enabled by sophisticated engineering of physicochemical properties, creating intelligent systems capable of context-aware interactions with disease microenvironments. The recent development of injectable hydrogel microspheres (HMPs) for cartilage repair exemplifies this transition toward multifunctional, localized regenerative platforms [7]. The design principles encompass complementary strategic approaches: first, surface functionalization with metabolites such as glucose analogs or lactate oxidase to mimic endogenous metabolic signaling while enhancing target cell engagement and minimizing systemic interference; second, incorporation of stimuli-responsive materials such as lactate-sensitive linkers and pH-triggered polymers to achieve release kinetics synchronized with disease-associated metabolic fluctuations such as glycolytic flux and acidosis; and third, optimization of physical parameters including size, elastic modulus, and topography to direct cellular metabolic responses through precise modulation of receptor clustering, endocytic pathways, and organelle organization [8–10]. This integrated engineering approach enables precise metabolic intervention in a spatiotemporally controlled manner.

The cooperative integration of these design elements enables the development of sophisticated therapeutic systems that can undergo context-dependent metabolic reprogramming. Surface-engineered microspheres with tailored physical characteristics demonstrate enhanced targeting and uptake efficiency, whereas microenvironment-responsive release permits the precise delivery of metabolic modulators in response to pathological signals such as lactate accumulation or adenosine triphosphate (ATP) depletion. Remarkably, the structural properties of microspheres can directly impose metabolic shifts through mechanobiological mechanisms, including cytoskeletal reorganization, altered substrate diffusivity, promotion of oxidative phosphorylation (OXPHOS), and suppression of glycolytic flux [11]. These complementary mechanisms operate synergistically to form adaptive systems that collectively recall metabolic networks through material-instructive and drug-mediated actions.

The expanding application landscape of metabolism-regulating microspheres demonstrates their therapeutic potential in pathologies where localized metabolic intervention is paramount. The strategic selection of OA, endometriosis, and diabetic retinopathy as examples spanning the musculoskeletal, reproductive, and visual systems serves to illustrate how fundamental microsphere strategies adapt to different metabolic landscapes and anatomical constraints. In the hypoxic avascular joint space of OA, microspheres are designed not only to correct dysregulated glycolytic flux but also to address the resultant lactic acidosis and aberrant lipid metabolism (e.g., abnormal fatty acid oxidation), which drive chondrocyte inflammation and cartilage breakdown [12]. Endometriosis is treated by regulating hormone levels (such as by reducing estradiol levels and down-regulating progesterone receptor expression) and overcoming physical barriers through in situ interventional delivery to target ectopic lesions [13]. Similarly, in diabetic retinopathy, periocular celecoxib-loaded poly(lactic-*co*-glycolic acid) (PLGA) microspheres correct diabetes-induced metabolic disturbances, resulting in reduced oxidative stress, diminished vascular leakage, and stabilization of the blood–retinal barrier [14]. These disparate applications effectively demonstrate how core strategies, including targeted delivery, microenvironment sensing (e.g., lactate or pH), and localized metabolic reprogramming (e.g., glucose and lipid utilization), can be universally applied for precise therapeutic intervention across disease contexts with shared metabolic features despite anatomical differences. This demonstrates versatility, establishing a conceptual and technical framework with broad applicability across numerous pathological conditions characterized by metabolic dysregulation, while also highlighting persistent challenges in tissue-specific targeting and biocompatibility that require further investigation.

**Fig. 1.**
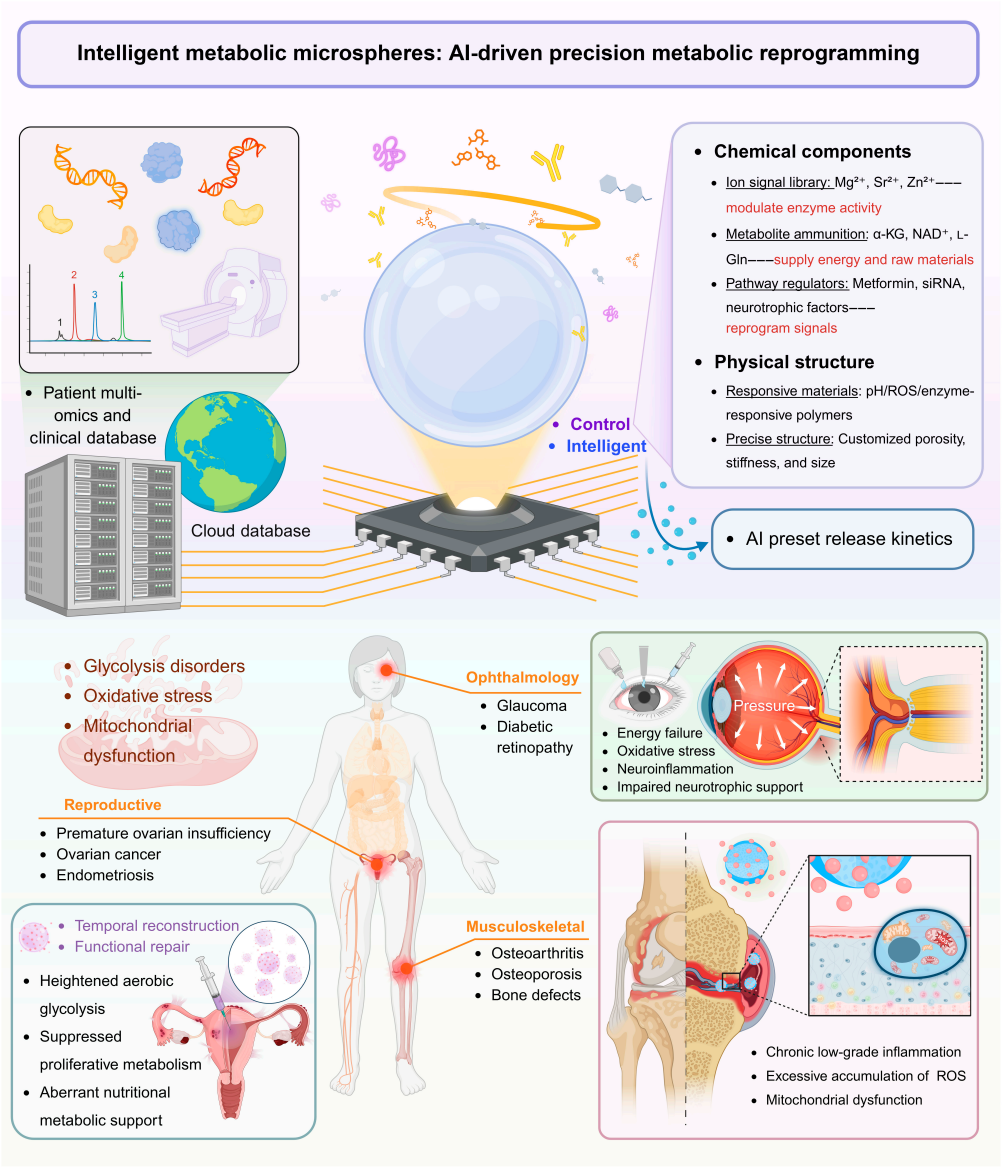
Schematic overview of intelligent metabolic microspheres for AI-driven precision metabolic reprogramming. The diagram illustrates the integration of patient multiomics data and a cloud-based chemical library to engineer microspheres with tailored chemical components and physical structures. These microspheres are designed to sense and respond to core-disease-specific metabolic alterations, such as glycolysis disorders, oxidative stress, and mitochondrial dysfunction, enabling temporally controlled release and functional repair in a range of tissues and pathologies, including OA, glaucoma, and endometriosis. siRNA, small interfering RNA.

This review systematically consolidates recent advances in metabolism-targeting microspheres, from fundamental material principles to clinical translation. By integrating insights from metabolic biology and biomaterials science, these engineered systems achieve unprecedented spatiotemporal control over local metabolic processes, which is a capability-reshaping treatment paradigm for degenerative, inflammatory, and metabolic diseases. As the field advances, crucial considerations regarding scalable manufacturing, biocompatibility optimization, and long-term safety validation require coordinated and interdisciplinary efforts. Subsequent sections critically examine these challenges alongside emerging solutions and chart promising trajectories for future research in this rapidly evolving field. Figure 1 provides a schematic overview of intelligent metabolic microspheres for artificial intelligence (AI)-driven precision metabolic reprogramming, illustrating their versatile therapeutic applications in pathological contexts such as OA, endometriosis, and glaucoma.

## Design Principles of Immunomodulatory Microspheres

The fabrication strategies for microspheres are pivotal in determining their structural stability, functional efficacy, and biosafety. These properties are key factors governing their applicability in advanced therapies that rely on metabolic reprogramming, such as controlled drug delivery for diseases including diabetes and tissue engineering for metabolic organs. Among the diverse preparation techniques, microfluidics, electrospraying, emulsification, and lithography have emerged as core methods [15]. Each offers unique capabilities for tailoring critical microsphere properties, including size, surface morphology, and drug-loading efficiency, which directly influence nutrient-sensing pathways and cellular metabolism. This precise control over the design has contributed to recent advancements in the development of intelligent metabolism-regulating biomaterials.

This microfluidic approach excels at generating uniformly sized and precisely structured microspheres. This method relies on the controlled manipulation of immiscible fluid phases, typically an aqueous phase containing hydrogel precursors and active agents and a surfactant-stabilized oil phase, in microfabricated channels. At the channel junction, shear forces and interfacial tension facilitate the formation of monodisperse droplets in the continuous oil phase. By adjusting parameters such as the flow rate ratio and channel geometry, researchers can precisely control the microsphere diameter from a few hundred micrometers and construct complex architectures such as core–shell or multilayered structures [16]. The principal advantage of microfluidics is its high reproducibility, which is critical for therapies that require strict batch-to-batch consistency, such as those aimed at maintaining a narrow therapeutic window for metabolic hormones.

In addition, the electrospray method uses electric-field-induced atomization for controlled microsphere production. A hydrogel precursor solution was injected through a nozzle under high voltage, forming a Taylor cone in which the jet disintegrated into uniform microdroplets owing to electrostatic repulsion. Subsequent solidification via chemical cross-linking or physical gelation yields stable microspheres [17]. A key advantage of electrospray is the facile tuning of microsphere size and morphology by modulating the voltage, flow rate, and nozzle diameter. This technique is compatible with various polymers and enables the gentle encapsulation of drugs (e.g., the chemotherapeutic doxorubicin), maintaining their bioactivity. It allows for the fabrication of microspheres with tunable release profiles, making it suitable for developing microspheres that target hepatocytes for precise metabolic intervention [18]. Despite these merits, their low production yield and stringent requirements for solution properties limit their scalability for industrial applications.

The emulsification technique—a mature and widely adopted process—is suitable for the large-scale manufacture of microspheres. In this method, an aqueous precursor solution is dispersed in a continuous oil phase via mechanical agitation or ultrasonic treatment, and surfactants are added to stabilize the resulting emulsion. The resulting droplets are subsequently solidified via cross-linking or gelation. Its notable advantages include a straightforward operation, low cost, and broad material compatibility (e.g., chitosan, collagen, and PLGA) [19]. This technique has been used to fabricate microspheres loaded with oral hypoglycemic drugs (such as metformin) that achieve sustained and controlled drug release and effectively maintain glucose homeostasis [20]. However, key limitations include a relatively broad particle size distribution and potential residual oil contamination, which may influence in vivo metabolic kinetics and biosafety.

Lithography is indispensable for producing metabolism-regulating microspheres with intricate geometries or customized surface topographies, which are critical for controlling drug release kinetics and interfacial interactions with metabolic tissues. This process begins with the creation of a photolithographic template containing predesigned microcavities. A hydrogel precursor solution is then cast into these molds and solidified via ultraviolet (UV) exposure or chemical cross-linking, resulting in microspheres with highly uniform and defined shapes [21]. A paramount advantage is the ability to precisely engineer geometric features, such as porosity or surface patterns, by modifying the template and enabling the design of microspheres that optimize nutrient diffusion, support the function of encapsulated metabolic cells, and enhance targeted delivery to specific metabolic organs [22].

**Fig. 2.**
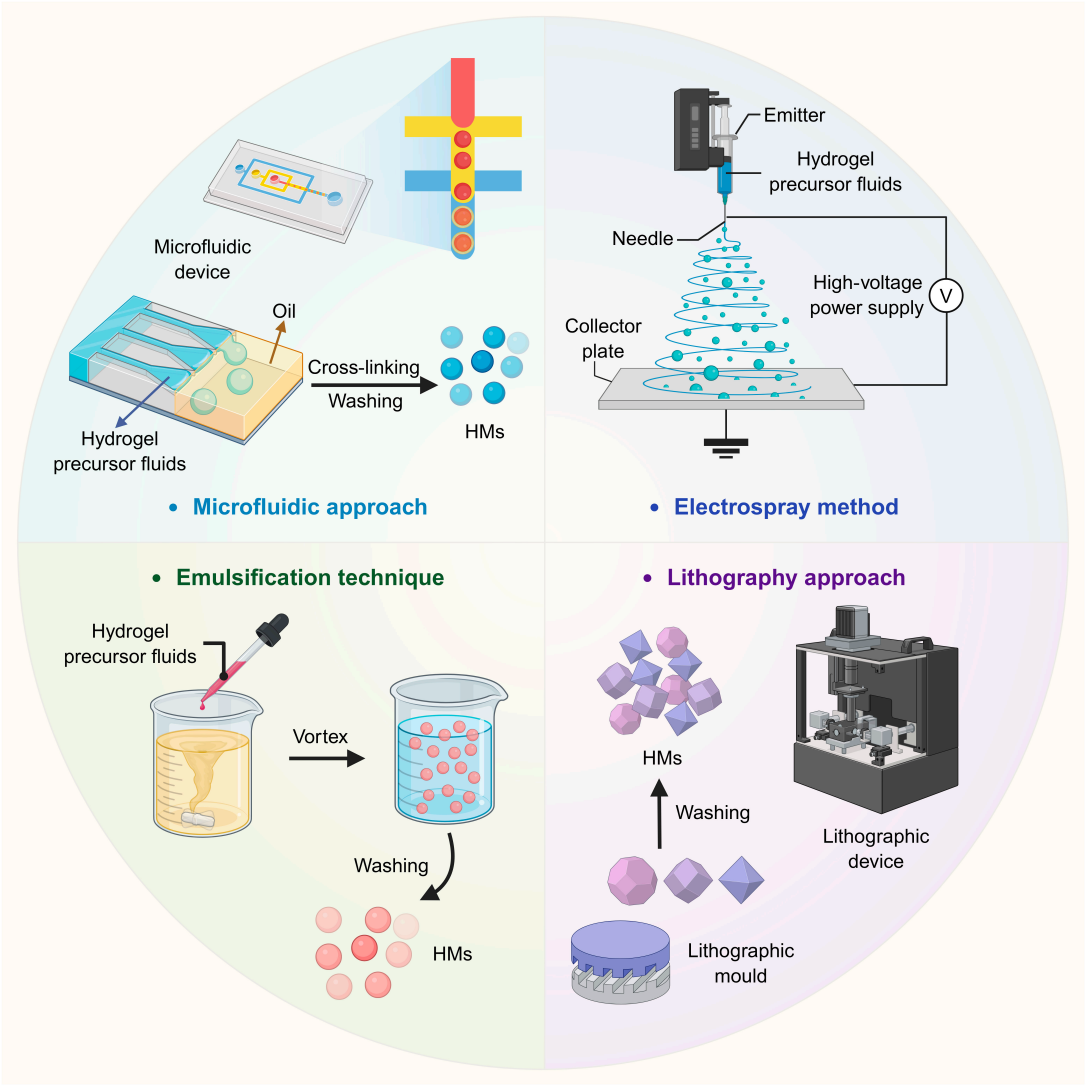
Schematic illustration of key fabrication methods for metabolism-regulating microspheres. Techniques such as microfluidics, electrospray, emulsification, and lithography enable precise control over the size, structure, and composition of microspheres.

The performance of microspheres is further governed by interdependent fabrication variables, including polymer selection, solvent properties, and reaction parameters. Strategic optimization, such as combining chitosan with alginate to enhance both mechanical stability and metabolic biocompatibility, can significantly improve critical functional attributes. Furthermore, hybrid strategies that synergistically integrate multiple techniques present a promising approach for overcoming the limitations of individual methods. In particular, for clinical translation, hybrid approaches that combine the precision of microfluidics with the scalability of emulsification have emerged as a highly viable scale-up pathway. For example, microfluidic chips based on step emulsification, equipped with hundreds of parallel nozzles acting as precision droplet generators, can form highly uniform emulsion templates. In such systems, the droplet size is primarily determined by the microchannel geometry and is relatively insensitive to flow rate fluctuations, thereby maintaining excellent monodispersity even in scaled-up production. Subsequently, these droplets can be solidified on a large scale via established processes such as UV polymerization, forming functionalized hydrogel microparticles [23]. This strategy preserves the advantages of microfluidics in structural uniformity and drug-loading control while achieving the high-throughput production capacity required for industrial and clinical manufacturing. It thereby provides a feasible technological platform for the large-scale preparation and translation of drug carriers. In summary, the strategic selection and refinement of fabrication approaches are central to the development of immunomodulatory microspheres. Each technique, from the precision of microfluidics to the scalability of emulsification, addresses specific requirements and enables the design of microspheres with tailored functions. Figure 2 summarizes the principal fabrication techniques, including microfluidics, electrospray, emulsification, and lithography, used to engineer microspheres that regulate metabolism with controlled size, structure, and functional properties.

## Metabolic Regulation Driven by Physicochemical Properties

**Fig. 3.**
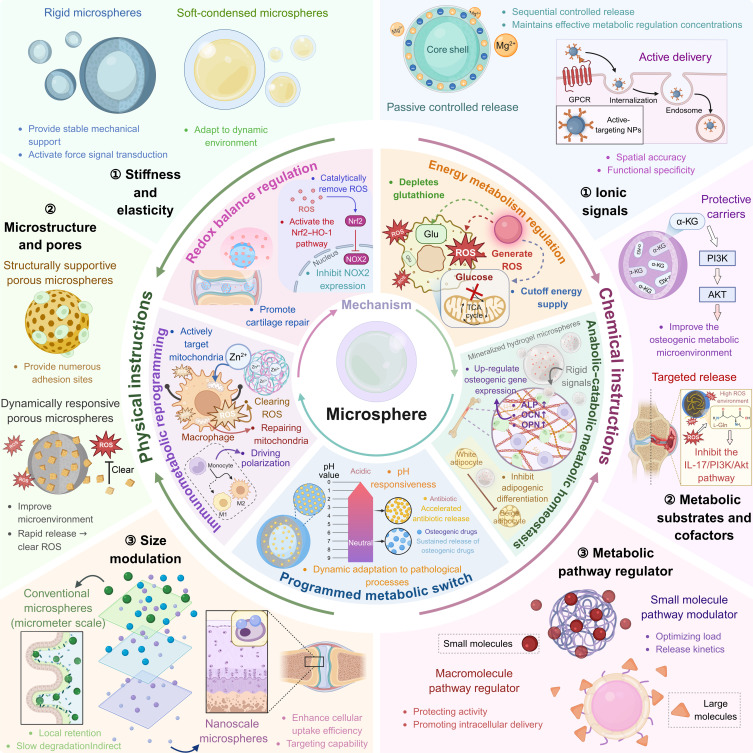
Schematic representation of metabolic regulation driven by the physicochemical properties of the microspheres. The chemical dimension orchestrates metabolic pathways by delivering ionic signals, metabolic substrates, cofactors, and pathway regulators. The physical dimensions directly influence cellular metabolic responses via engineered stiffness and elasticity, tailored microstructure and porosity, and controlled size. Together, these properties enable microspheres to precisely regulate key metabolic processes, including energy metabolism, redox balance, immunometabolic reprogramming, anabolic–catabolic balance, and temporal adaptation to the pathological microenvironment. GPCR, G-protein–coupled receptor; NOX2, reduced form of nicotinamide adenine dinucleotide phosphate oxidase 2; ALP, alkaline phosphatase; OCN, osteocancin; OPN, osteopontin.

Metabolically active microspheres have advanced from conventional carriers to integrated platforms that combine passive nutrient delivery with active metabolic regulation for precise physiological modulation. Achieving targeted metabolic interventions without systemic disruption requires a synergistic design incorporating 2 fundamental dimensions: the chemical dimension, which orchestrates metabolic signaling pathways, and the physical dimension, which regulates cellular metabolic recognition. Microspheres program metabolic responses through 3 primary mechanisms: ion signaling for enzymatic modulation, provision of metabolic substrates and cofactors, and metabolic pathway modulation. Physically, they guide cellular processes via engineered parameters, such as stiffness, tailored microstructure, porosity, and size-dependent distribution patterns. As schematically summarized in Fig. 3, the metabolic reprogramming capabilities of the microspheres are driven by an integrated design of chemical and physical cues. The chemical dimensions use ionic signals, metabolic substrates, cofactors, and pathway regulators, whereas the physical dimensions use stiffness and elasticity, microstructure and porosity, and size adjustment. These elements collectively enable precise regulation of diverse metabolic processes.

## Metabolic Modulation Driven by Chemical Properties

In metabolism-regulating microspheres, the precise encoding of chemical instructions is central to directing cellular fate. Through tailored composition and loading strategies, bioactive signals—ions, metabolic substrates/cofactors, and pathway regulators—are transformed into a “chemical language” that can be interpreted by cells. These instructions actively and directionally modulate intracellular metabolic flux, energy status, and signal transduction networks, guiding cellular behavior toward therapeutic outcomes. Ions, substrates/cofactors, and regulators function synergistically at 3 levels: rapid signal initiation, homeostatic maintenance, and deep-layer network reprogramming, forming a structured toolkit for precision tissue regeneration therapy and disease treatment.

### Ionic signals

Ionic signals function as crucial “metabolic instructions”, with their efficacy depending on the microsphere delivery strategy. Rational composition and structural design enable either passive controlled release or active delivery, allowing precise intervention in processes such as the immune response, osteogenesis, and angiogenesis.

In passive strategies, microspheres leverage their material composition and degradation properties for the temporal release of ions. For instance, in PLGA/MgO-alginate core–shell microspheres, the alginate shell acts as a degradable barrier, controlling the stable release of Mg^2+^ at ~50 parts per million/d for over 2 weeks to activate osteogenic pathways [24]. Similarly, PLGA/MgO/MgCO_3_ microspheres coembed fast- and slow-release components, enabling “programmable” Mg^2+^ kinetics to optimize osteogenic differentiation [25].

In active strategies, microspheres use chemical modifications or smart elements to transform from passive “ionic reservoirs” into active “metabolic interveners”. The FM@CeZnHA system uses triphenyl phosphonium to target cerium–zinc-doped nanoparticles to macrophage mitochondria, directing Ce^3+^/Ce^4+^ and Zn^2+^ ions to scavenge reactive oxygen species (ROS) and repair mitochondrial networks, reprogramming glucose metabolism at its source [26]. Gelatin methacryloyl (GelMA)-biphosphonate (BP)-Mg-nanobubble (NB) ultrasound-responsive microspheres couple Mg^2+^ release to external ultrasonic stimulation via nanobubble cavitation, achieving an on-demand, “blast-like” release that activates osteogenic pathways at critical time points [27].

### Metabolic substrates and cofactors

Metabolic substrates and cofactors serve as fundamental chemical instructions for precise metabolic interventions. The design of microspheres ensures stable encapsulation, protection, and delivery via controlled-release mechanisms, directly regulating energy metabolism, redox balance, and immune polarization to reshape the metabolic microenvironment.

Passive sustained-release systems function as protective carriers. Calcium phosphate bone cement/α-ketoglutarate (α-KG) polyester microspheres provide a long-term supply of α-KG, a tricarboxylic acid (TCA) cycle intermediate and prolyl hydroxylase domain protein 2 (PHD2) cosubstrate, to scavenge ROS, inhibit inflammation, and activate the phosphatidylinositol 3-kinase (PI3K)/AKT pathway in osteoporotic bone defects [28]. Nicotinamide riboside (NR)/resveratrol self-assembled microspheres use pH-responsive release to protect NR for efficient intestinal absorption, elevating systemic nicotinamide adenine dinucleotide (oxidized form) (NAD^+^) levels, and ameliorating mitochondrial energy metabolism in myocardial injury [29].

Active delivery systems address challenges such as cellular uptake barriers and spatiotemporal specificity. The nicotinamide adenine dinucleotide-loaded@nanoparticles@hydrogel microsphere (NAD@NPs@HM) system uses lactoferrin-modified liposome–HMPs for efficient NAD delivery, enhancing endocytosis to elevate intracellular NAD, reversing cellular senescence, and promoting M2 macrophage polarization [30]. The l-glutamine@gelatin microspheres@3-carboxyl-4-fluoroboric acid-grafted quaternized chitosan and polyvinyl alcohol composite microspheres encapsulate l-glutamine in an ROS-responsive hydrogel for targeted release in osteoarthritic joints, correcting the anabolic–catabolic imbalance in chondrocytes and regulating macrophage metabolism [31].

### Metabolic pathway regulators

Metabolic pathway regulators are key molecules that directly regulate cellular signal transduction and metabolic pathways. Their efficacy depends on the precise delivery of the microspheres. Unlike substrates or ions, these regulators actively reprogram metabolic states by targeting specific enzymes, receptors, and transcription factors.

Microspheres enhance the duration and targeting of small-molecule regulators. Metformin@sulfobetaine-loaded hyaluronic acid methacrylate (Met@SBHA) microspheres use betaine-modified hyaluronic acid for sustained Met release and joint lubrication, synergistically delaying chondrocyte senescence via the inducible nitric oxide synthase/ONOO^−^/p53 pathway [32]. Zirconium–tetrakis(4-carboxyphenyl)porphyrin@siFOXK2@cell membrane/metformin@gelatin methacryloyl (ZrTCP@siFOXK2@CM/Met@GelMA) composite microspheres codeliver Met and siFOXK2, inhibit 5′-adenosine monophosphate-activated protein kinase, and silence proglycolytic FOXK2 when supplemented with photodynamic therapy to disrupt the Warburg effect in ovarian cancer [33].

For macromolecular regulators (proteins and nucleic acids), microspheres prioritize the preservation of bioactivity and intracellular delivery. Polyethyleneimine–phenylboronic acid–bone morphogenetic protein 2 (BMP-2)–adipose-derived stem cell HMPs use dynamic covalent chemistry to control BMP-2 release and codeliver stem cells to repair critically sized bone defects [34]. Rapamycin-encapsulated exosome-mimetic nanoparticles-in-microspheres use a 2-stage delivery strategy to enhance cellular uptake and prolong release, improving cytotoxicity against hemangioma stem cells [35]. Chitosan microspheres function as prebiotics to remodel the gut microbiota and indirectly regulate host liver microRNAs (miRNAs) (e.g., miR-103) to ameliorate glycolipid metabolic disorders in diabetes [36].

The precise encoding of chemical commands in microspheres enables active metabolic intervention. Future advancements will require the temporal sequencing of these commands, dose modulation, and deeper synergy with physical properties to simulate an in vivo biochemical–physical coupled microenvironment.

## Metabolic Modulation Driven by Physical Properties

The paradigm of metabolism-regulating microspheres has shifted from passive drug depots to active cellular interventions, with the recognition that cellular metabolism is profoundly influenced by the physical microenvironment. The intrinsic physical properties of microspheres (stiffness, elasticity, microstructure, porosity, and size) constitute a set of critical “physical instructions”. These instructions directly trigger mechanobiological responses, reprogram cellular metabolism by altering energy pathways, modulate anabolism/catabolism, and influence immune polarization, enabling diverse therapeutic outcomes.

### Stiffness and elasticity

Stiffness and elasticity are core physical properties that directly regulate cellular metabolism. Stiffness determines the structural stability and mechanical signal transmission, whereas elasticity enables adaptation to dynamic environments. Microspheres guide metabolism by mimicking the mechanics of native tissues. Microspheres can be categorized into rigid microspheres, which promote anabolic processes such as osteogenesis, and soft-condensed microspheres, which regulate processes such as tumor suppression via low stiffness and high elasticity.

Rigid microspheres provide stable mechanical support. Mineralized HMPs, with a compressive modulus of 15.65 kPa, match the bone microenvironment and up-regulate osteogenic genes (*Runx2* and *OCN*) in bone marrow mesenchymal stem cells (BMSCs) to steer metabolism toward bone formation [37]. PLGA microspheres in poly(methyl methacrylate) cement (elastic modulus, 533.4 MPa) avoid stress shielding while providing mechanical anchors that promote osteogenic and angiogenic gene expression [38]. In soft tissues, polycaprolactone and amino-modified poly-L-lactic microspheres act as long-term “mechanical scaffolds”, activating the transforming growth factor-β/Smad pathways in fibroblasts to up-regulate collagen synthesis and counteract metabolic decline [39].

Soft-condensed microspheres adapt to deformation. Microspheres, with a low storage modulus (90 to 100 Pa) and high elasticity, are densely packed in the tumor vasculature, creating sustained physical compression to block nutrient supply and suppress tumor metabolism for over a month [40]. Polyethylene glycol (PEG)–fibrinogen breast cancer microspheres have low initial stiffness (45 ± 5 Pa) that cells can remodel; MCF7 cells increase stiffness to 172 Pa, promoting proliferative metabolism, whereas MDA-MB-231 cells maintain stable metabolism, demonstrating how microsphere mechanics regulate tumor cell phenotypes [41].

### Microstructure and porosity

The microstructure and pore characteristics (size, porosity, connectivity, and hierarchy) are core physical factors that determine the specific surface area, transport efficiency, and cell–material interactions. They create a biomimetic physical microenvironment primarily through structurally supportive and dynamically responsive porous microspheres.

Structurally supportive porous microspheres establish stable frameworks for long-term cell habitation and material exchange. Brachyury mRNA-loaded lipid nanoparticle@gelatin methacryloyl/fucoidan dual-component hydrogel microspheres offer uniform pores for nucleus pulposus cell adhesion and distribution, thus enhancing metabolic activity [42]. Porous starch microspheres have a hierarchical structure in which macropores allow enzyme diffusion and mesopores create steric hindrance, physically delaying digestive metabolism [43].

Dynamically responsive porous microspheres adjust their pore structures to pathological signals for on-demand metabolic interventions. GMPB (gelatin methacryloyl/glycosaminoglycan-derivative ChsMA hydrogel microspheres integrated with Prussian blue nanozymes and bone marrow mesenchymal stem cells) microspheres use interconnected pores for rapid ROS-scavenging nanozyme release early in degeneration, which later stabilizes to support stem cell paracrine signaling for long-term anabolic metabolism [44]. Vancomycin-loaded chitosan microspheres/diflunisal-loaded chitosan microspheres feature pH-responsive chitosan pores that expand in acidic, infected environments to accelerate vancomycin release and then contract at neutral pH for sustained diflunisal release to activate osteoblast metabolism [45].

### Size adjustment

Size modulation is a critical physical parameter that influences in vivo distribution, cellular uptake, and metabolic outcomes. Nanoscale microspheres (<1 μm) target sites via the enhanced permeability and retention effect, are readily internalized, and enable intracellular metabolic reprogramming. Conventional microspheres (micrometer scale, >1 μm) serve as localized sustained-release platforms or scaffolds, modulating the microenvironment through physical retention.

Nanoscale microspheres enable precise intracellular interventions. Outer membrane vesicle–AgBiS_2_@ICG microspheres (~166 nm) penetrate periodontal biofilms, are internalized by bacteria, and disrupt bacterial energy metabolism and synthesis pathways [46]. WYGRL peptide-modified Au@Pt microspheres (~56.9 nm) accumulate in chondrocytes via a targeting peptide, clearing ROS and activating the nuclear factor erythroid-2-related factor 2–heme oxygenase-1 pathway to promote cartilage repair [47]. MoO/Fe@PEG microspheres (50 to 80 nm) deplete glutathione and generate ROS in tumor cells, disrupting the redox balance and glucose metabolism for closed-loop metabolic regulation [48].

Conventional microspheres regulate local retention and sustained release. Macrophage membrane-coated SeNPs@HAMA hydrogel microspheres (~219 μm) distribute uniformly in joints, degrading to sustainably release selenium-containing nanoparticles that regulate macrophage polarization and ameliorate chondrocyte matrix imbalance [49]. PLGA@MiR&COD microspheres (~2.58 μm) are retained in peritoneal tumors, releasing miriplatin (MiR) and cholesterol oxidase (COD) to disrupt membrane integrity and mitochondrial function, reversing drug resistance metabolism [50].

The synergistic interplay between chemical and physical instruction sets is exemplified in the design of injectable HMPs. A representative system involves chondroitin sulfate microspheres encapsulating a gallic acid–magnesium metal–organic framework loaded with puerarin. In this design, physical instructions are primarily delivered by the microsphere scaffold itself. Hydrogel network functions as a protective depot, physically confining therapeutic agents to enable localized, extended release over weeks, thus setting a permissive physical context for long-term chemical signaling. Within this stable physical context, a sequential chemical instruction set is executed to reprogram chondrocyte metabolism from a catabolic, inflammatory state toward an anabolic, homeostatic phenotype. The degradation of the microspheres orchestrates a timed release. First, gallic acid scavenges ROS and exerts anti-inflammatory effects, rapidly ameliorating the hostile metabolic microenvironment. Subsequently, magnesium ions are released, up-regulating hypoxia-inducible factor-1α (HIF-1α) expression in chondrocytes to promote the synthesis of cartilage matrix components such as type II collagen [51]. This OA-targeted example demonstrates the proposed hierarchy: Physical cues establish the essential, permissive stage, enabling and enhancing the efficacy of the sequentially delivered chemical commands that directly shift metabolic flux from a catabolic, inflammatory state toward an anabolic, homeostatic one.

The stiffness/elasticity, microstructure/porosity, and size of microspheres form a powerful “physical instruction set” for direct metabolic regulation. Rigid microspheres guide anabolism, soft-condensed microspheres adapt to suppress tumors, porous structures optimize exchange, and their size dictates the intervention strategy. These aspects synergistically establish a rational design foundation for metabolism-regulating microspheres.

## Therapeutic Application of Metabolic Microspheres for Orthopedic Diseases

Skeletal homeostasis and repair are biologically regulated processes that are driven by cellular metabolism. Under conditions such as osteoporosis (OP), OA, and nonhealing bone defects, this process is frequently disrupted by energy metabolism imbalances and self-perpetuating inflammatory cycles. Traditional therapeutic strategies, hampered by the “targeting inaccuracies” of systemic administration or the “single-function limitations” of conventional biomaterials, struggle to achieve precise multitarget interventions at the core of these pathologies. The emergence of metabolically regulated microspheres marks a paradigm shift in orthopedics from “passive replacement” to “active regeneration”. These microspheres function not only as drug delivery vehicles but as integrated “metabolic regulators”, “ionic signaling hubs”, and “niche coordinators”. Through their functional components and responsive structures, they synergistically regulate the osteoblast–osteoclast balance, promote angiogenesis, and clear pathological metabolites, systematically remodeling the local metabolic microenvironment. This section explores the application of these microspheres in 3 major orthopedic diseases, analyzes how innovations in material science enable precise metabolic remodeling, and discusses their potential to advance orthopedic therapy into a new era of physiological repair.

## Osteoarthritis

OA is now recognized as a metabolic dysfunction rather than merely mechanical wear. The OA joint has a complex pathological microenvironment characterized by chronic low-grade inflammation, excessive ROS production, and mitochondrial dysfunction. These factors disrupt chondrocyte metabolic homeostasis, leading to insufficient mitophagy and regulated cell death pathways, such as ferroptosis, ultimately causing an imbalance in extracellular matrix (ECM) synthesis and degradation [52]. Current treatments, such as nonsteroidal anti-inflammatory drugs and hyaluronic acid injections, offer symptomatic relief without reversing the disease course. Metabolism-regulating microspheres function as engineered platforms that target the metabolic root causes of OA, shifting the strategy from “symptom management” to “etiological intervention”.

**Fig. 4.**
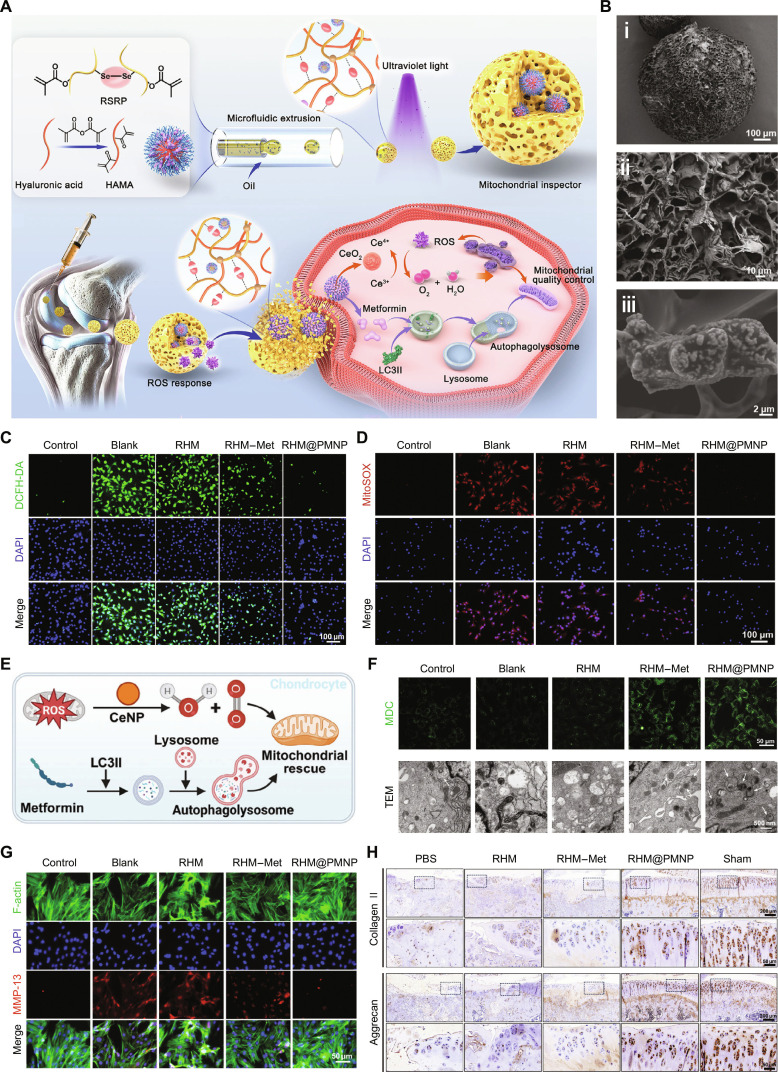
The mitochondrial-regulating microsphere system constructed via dynamic diselenide bonds alleviates OA by scavenging ROS, activating autophagy, and preserving the cartilage ECM. (A) Synthesis of ROS-sensitive selenide-containing polymer (RSRP) by incorporating dynamic diselenide bonds enables the construction of a “mitochondrial inspector” to treat OA via mitochondrial quality control. HAMA, methacrylated hyaluronic acid; LC3II, light chain 3-II. (B) Scanning electron microscopy (SEM) examination of ROS-responsive hydrogel microspheres@PAA-modified CeNPs and metformin nanoparticle (RHM@PMNP) from (i) a broad view and (ii and iii) a close-up view. (C) ROS production was measured using dichlorofluorescein-diacetate (DCFH-DA). DAPI, 4′,6-diamidino-2-phenylindole. (D) Mitochondrial ROS generation was analyzed using MitoSOX. (E) Illustration of the process by which RHM@PMNP controls the cell homeostasis. (F) Monodansylcadaverine (MDC) staining results and biological transmission electron microscopy (TEM) observation of mitochondria with autophagic vesicles, indicated by white arrows, collectively confirmed autophagy activation. (G) Immunofluorescence staining for MMP-13. (H) Immunohistochemical staining for collagen II and aggrecan collectively demonstrated the synthesis of the cartilage ECM. PBS, phosphate-buffered saline. This figure has been reproduced with permission. Copyright 2025, Wiley-VCH.

In OA treatment, these microspheres function as “smart regulators” when implanted in the joint cavity, directly intervening in chondrocyte energy metabolism and in redox homeostasis [53]. For instance, cerium dioxide nanozyme-containing microspheres act as persistent catalytic centers, mimicking superoxide dismutase/catalase activities within the high-ROS microenvironment of the joint. These microspheres efficiently scavenge ROS, significantly reducing both total intracellular ROS and mitochondrial superoxide levels in OA chondrocytes to near-normal ranges. This amelioration of the oxidative stress microenvironment, in synergy with metformin, activates autophagy to clear dysfunctional mitochondria, resulting in a restoration of the mitochondrial membrane potential by over 20-fold. Ultimately, this mitochondrial quality control strategy effectively maintains cellular homeostasis: It significantly suppresses the expression of the matrix-degrading enzyme MMP-13 (matrix metalloproteinase 13) while promoting the synthesis of type II collagen and aggrecan, thereby demonstrating the potential of the microspheres to mitigate OA progression, as illustrated in Fig. 4 [54]. In parallel, a “Trojan horse”-inspired microsphere system (cationic targeting nanoparticle-hydrogel microspheres@fucoidan) achieves deep cartilage targeting and sustained release of fucoidan, which enhances mitochondrial energy production and counters cellular senescence to promote regeneration [55]. Similarly, microspheres with intelligently responsive diselenide bonds function as “metabolic waste sensors”, where ROS-triggered cleavage of the polymer backbone precisely initiates drug release, ensuring on-demand and efficient clearance of metabolic waste [56].

Besides immediate sensing and clearance, microspheres act as long-term metabolic resource managers. To counter the widespread selenium deficiency in OA, microspheres loaded with selenium nanoparticles serve as a sustained-release reservoir, continuously supplying this critical trace element to support the synthesis of endogenous antioxidant selenoproteins, such as glutathione peroxidase (GPX), and rebuilding the long-term defense system of chondrocytes [57]. To combat ferroptosis, curcumin-loaded functionalized microspheres exploit curcumin’s β-diketone groups to actively chelate and sequester free Fe^2+^, physically blocking the Fenton reaction and lipid peroxidation as proactive “scavengers” of harmful substances [58].

## Osteoporosis

OP is a systemic skeletal disorder characterized by low bone mass and deteriorated microarchitecture, rooted in a metabolic imbalance in which bone resorption outpaces bone formation. This involves not only dysfunctional osteoclast–osteoblast coupling but also broader physiological disturbances, such as aberrant cellular energy metabolism, which collectively impair the self-repair capacity of the skeleton. Current pharmacological therapies are limited by poor targeting specificity and the inability to maintain long-term effective local concentrations. Metabolism-modulating microspheres have been engineered to overcome these limitations by providing bone-targeting and sustained-release capabilities, enabling localized and prolonged modulation of the bone microenvironment to correct local bone loss.

Therefore, precise drug delivery is essential for effective metabolic regulation. The POSS-DFO-Asp8-PEG@alginate/chitosan microspheres use a dual-targeting design for oral and bone-specific delivery, accurately transporting active molecules to bone tissue. This system concurrently promotes type H vessel formation and suppresses osteoclast activity, synergistically addressing bone loss by improving nutrient supply and reducing resorption [59]. In contrast, salmon calcitonin-loaded size-switchable microspheres@anhydrous reverse micelle microspheres optimize the pharmacokinetics of salmon calcitonin (sCT). By encapsulating sCT within anhydrous reverse micelles (ARMs) embedded in a PLGA/poly(cyclohexane-1,4-diylacetone dimenthylene ketal) matrix, they create a dual-protection system that shields sCT from enzymatic degradation and extended its release to 16 d. This resolves the clinical challenge of sCT’s short half-life by maintaining stable therapeutic plasma levels to continuously activate calcitonin receptors on osteoclasts and suppress bone resorption [60].

Building on precise delivery, various microsphere systems can be used to target specific nodes within the bone metabolic network for the physiological restoration. CS–SrCSH microspheres use an ionic homeostasis strategy, wherein sustained Sr^2+^ release activates the mitogen-activated protein kinase (MAPK)/extracellular signal–regulated kinase (ERK) pathway in osteoblasts, promoting their proliferation and differentiation. Simultaneously, Sr^2+^ instructs osteoblasts to up-regulate osteoprotegerin, which acts as a decoy receptor to neutralize receptor activator of nuclear factor κB ligand (RANKL) and suppress osteoclastogenesis at its source [61]. Conversely, GelMA-AS-ligustrum polysaccharide (LOP) microspheres adopt a multitarget approach. Codelivered alendronate is internalized by osteoclasts to inhibit the MAPK/nuclear factor of activated T cells, cytoplasmic 1 (NFATc1) axis, whereas LOP activates the PI3K/Akt pathway in osteoblasts. This coordinated design bidirectionally suppresses osteoclast activity and enhances osteogenesis [62].

## Bone Defects

Impaired healing of large bone defects stems from structural damage and severe metabolic microenvironmental imbalance. This pathological state is characterized by hypoxia-induced metabolic reprogramming and a self-reinforcing inflammatory cycle driven by glycolysis, ultimately leading to the complete stagnation of regeneration. Traditional biomaterials can only provide passive support and fail to actively correct pathological states. In recent years, “active-type” repair strategies that release bioactive components through materials to regulate cellular metabolism have demonstrated significant potential [63]. However, these bulk materials inherently lack spatiotemporal precision for metabolic regulation. Consequently, “metabolic regulation microspheres” capable of controlled and timed signal release have emerged as a research focus for next-generation biomaterials.

These microspheres achieve multidimensional interventions at key metabolic nodes. For instance, Ce/Zn codoped mitochondria-targeted microspheres release metal ions that localize to macrophage mitochondria, scavenge ROS, and restore mitochondrial function. This shifts macrophage metabolism from glycolysis to OXPHOS, driving the transition from a proinflammatory (M1) to a proreparative (M2) phenotype for precise immunometabolic reprogramming [26]. Alternatively, microspheres can act as critical metabolic substrate sources. α-KG polyester microspheres sustainably release this TCA cycle intermediate, directly fueling osteoblast mitochondrial OXPHOS for enhanced ATP production while activating the PI3K/Akt pathway to overcome the local “energy crisis” [28]. Furthermore, tannic-acid-modified alginate/chitosan microspheres demonstrated dual regulation by suppressing the RANKL–NFATc1 pathway in osteoclasts and activating runt-related transcription factor 2 in osteoblasts, directly coordinating the metabolic balance between resorption and formation [64].

They also function as hubs for delivering endogenous bioactive signals. By covalently immobilizing young ECM (Y-ECM) onto their surface, they construct a “rejuvenating” microenvironment. These functionalized microspheres deliver Y-ECM-derived signals, including mitochondrial proteins, to senescent stem cells, significantly up-regulating sirtuin 3 expression to enhance mitochondrial function and biosynthesis, reversing metabolic decline [65]. Microspheres amplify cell-derived signals in vascularized bone regeneration strategies. When combined with Ets variant transcription factor 2-overexpressing stem cells, hydroxyapatite/chitosan microspheres enhance the sustained release of metabolically active factors (e.g., vascular endothelial growth factor [VEGF] regulated via the PHD2–HIF-1α axis), synergistically optimizing energy metabolism for concurrent angiogenesis and osteogenesis [66].

Looking forward, the true potential of these intelligent biomaterial systems as “metabolic command systems” may be further realized through strategic synergy with existing clinical modalities. For instance, multifunctional metabolism-regulating microspheres could be engineered to provide localized, sustained delivery of approved anabolic agents (such as teriparatide or its analogs) [67]. This approach would aim to overcome the limitations of systemic administration, such as a narrow therapeutic window and the need for frequent injections. By maintaining optimal drug concentrations directly within the bone defect site, this strategy could amplify osteogenic signals while minimizing off-target effects. Furthermore, such microsphere platforms can serve as ideal scaffolds and signaling hubs for adjunctive cell therapies. By preprogramming their functions to sequentially provide metabolic support (e.g., mitigating initial oxidative stress, supplying osteogenic ions, and coreleasing immunomodulatory signals), they would create a hospitable and instructive niche. This niche could significantly enhance the survival, engraftment, and therapeutic function of codelivered mesenchymal stem cells or osteoprogenitors [68].

Metabolic regulation microspheres are driving a paradigm shift in orthopedics from “passive support” to “active metabolic intervention”. In OP, they act as precise messengers and functional restorers, systematically correcting the osteogenesis-osteoclastogenesis imbalance. In OA, they function as catalytic centers or clearance units, directly reshaping chondrocyte redox and energy homeostasis. In bone defect repair, they function as temporal metabolic command systems that actively regulate immunity, energy, and stem cell metabolism to drive regeneration. Despite their various applications, their core mechanism lies in using designed structures, responsive release, and active components that directly intervene at the cellular metabolic level. The future lies in the development of intelligent systems capable of dynamically sensing pathological cues and programmatically releasing multimodal commands. This evolution from “symptomatic treatment” to “etiological reconstruction” paves a promising pathway for restoring skeletal physiological functions.

## Therapeutic Strategy of Metabolic Microspheres for Ophthalmic Diseases

Ophthalmic diseases, including glaucoma and diabetic retinopathy, exhibit distinct pathological manifestations and share a common underlying driver: disrupted metabolic homeostasis in the retinal tissues. These disturbances encompass interconnected pathways, such as energy failure, oxidative stress, neuroinflammation, and impaired neurotrophic support, which collectively contribute to progressive and irreversible neuronal damage [69]. Conventional clinical approaches, which are largely focused on managing isolated symptoms or risk factors, face inherent pharmacological limitations, including pulsatile drug release, poor bioavailability, and a lack of tissue specificity. These limitations hinder the provision of sustained, stable, and multilevel metabolic support necessary to reverse neurodegenerative processes. This shared therapeutic gap has accelerated the demand for a new generation of interventional strategies. In this context, metabolically regulated microspheres have emerged as transformative platforms that transcend the role of traditional drug carriers. They integrate prolonged controlled release, multitarget synergy, and lesion-specific delivery into a single system. Through sophisticated design, these microspheres align drug pharmacokinetics with the chronic and multifactorial nature of ophthalmic diseases. They extend therapeutic coverage over weeks to months and spatially and functionally coordinate drugs with complementary mechanisms, establishing an integrated “metabolic regulation network” at the disease site.

## Glaucoma

Glaucoma is a progressive neurodegenerative disease characterized by the irreversible loss of retinal ganglion cells (RGCs). Although elevated intraocular pressure (IOP) is a key risk factor, it is not the sole driver; a disrupted metabolic microenvironment surrounding RGCs critically contributes to apoptosis. This disruption involves multiple metabolic crises: impaired mitochondrial energy metabolism in RGCs because of obstructed axonal transport, activated glial cells that adopt a proinflammatory phenotype and compete with RGCs for glucose, and breakdown of the metabolic support network because of disrupted neurotrophic signaling [70]. Conventional therapies primarily target IOP reduction and are largely ineffective in treating complex metabolic imbalances. Furthermore, traditional eye drops suffer from low bioavailability, poor compliance, and pulsatile dosing, which fail to provide the sustained metabolic support required for neuroprotection.

**Fig. 5.**
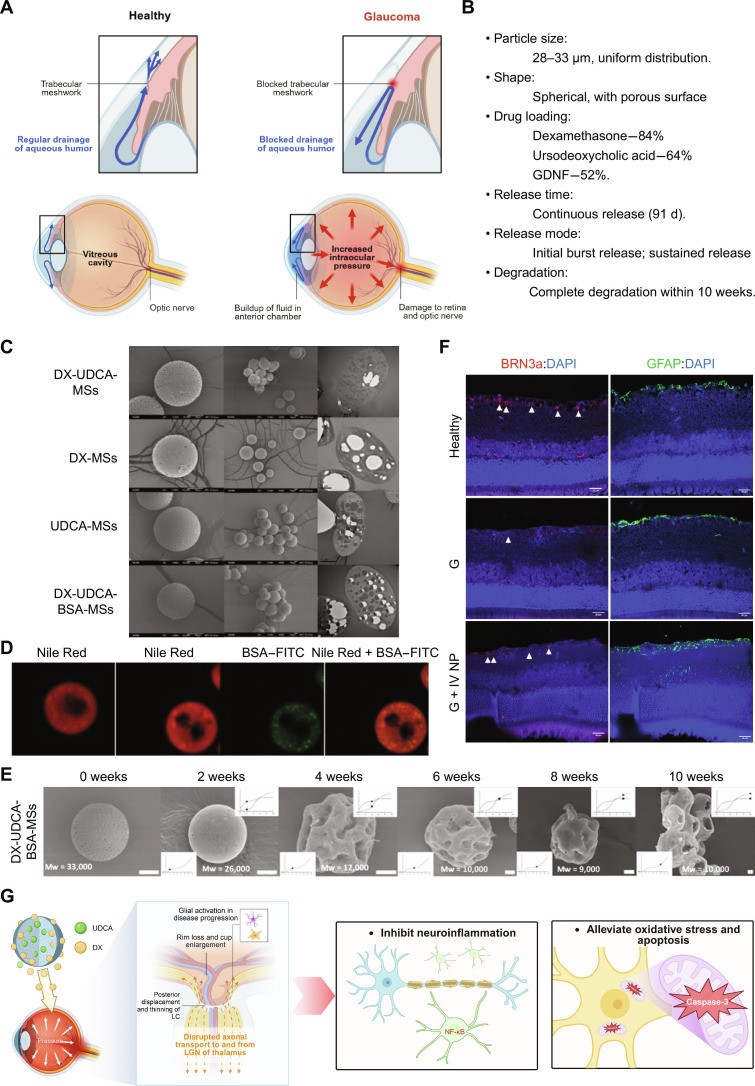
The multiloaded PLGA microsphere platform codelivers anti-inflammatory dexamethasone, antiapoptotic ursodeoxycholic acid, and neurotrophic glial-cell-line-derived neurotrophic factor (GDNF) to synergistically restore the RGC metabolic microenvironment, demonstrating sustained-release kinetics and significant neuroprotective efficacy in a chronic glaucoma model. (A) The diagram contrasts normal aqueous humor drainage through the trabecular meshwork in healthy eyes with its blockage and the resultant elevation of IOP in glaucoma. (B) Summary of the key physicochemical and drug release properties of a microsphere-based platform designed for sustained multidrug delivery in glaucoma treatment. (C) SEM and TEM images and particle size distribution of the selected formulation (dexamethasone and ursodeoxycholic acid coloaded poly[lactic-*co*-glycolic acid] microspheres [DX-UDCA-MSs]), the only loaded DX formulation (DX-MSs), the only loaded ursodeoxycholic acid (UDCA) formulation (UDCA-MSs), and the selected formulation with protein included (DX-UDCA-GDNF-MSs) MSs. Individual scanning pictures were taken at ×2,000, and group pictures were taken at ×500. (D) Confocal microscopy images of Nile-Red-labeled PLGA microspheres and bovine serum albumin (BSA)–fluorescein isothiocyanate (FITC) distribution. From left to right: Microspheres of formulation DX-UDCA-MSs, microspheres of formulation DX-UDCA-GDNF-MSs replacing GDNF with BSA–FITC (Nile Red channel, BSA–FITC channel, and combination of Nile Red and BSA–FITC channels). (E) SEM images showing the degradation process of DX-UDCA-BSA-MSs over time (0, 2, 4, 6, 8, and 10 weeks) and the corresponding changes in PLGA molecular weight (Mw). Scale bars, 10 μm. (F) Histological analysis of retinas at 24 weeks postinjection. Comparison of RGC counts (brain-specific homeobox/POU domain protein 3A [Brn3a] staining; left column) and astrogliosis (glial fibrillary acidic protein [GFAP] staining; right column) in the 3 cohorts. Healthy cohort noninjected; G, glaucoma; G + IV NP, glaucoma + neuroprotection. (G) Glaucoma pathophysiology and multitarget therapeutic mechanisms of UDCA and DX in inhibiting neuroinflammation, alleviating oxidative stress, and reducing apoptosis to protect ganglion cells. LGN, lateral geniculate nucleus; NF-κB, nuclear factor κB. This article is licensed under the Creative Commons Attribution 4.0 International License (CC BY 4.0).

Metabolically regulatory microspheres overcome these limitations by enabling long-acting, synergistic, and precise regulation of the RGC microenvironment. Functioning as a versatile platform, they can integrate various active ingredients, such as anti-inflammatory agents, mitochondrial protectants, and neurotrophic factors, to establish a spatiotemporally coordinated “metabolic regulation network” [71]. Coloading these components into a single microsphere system avoids repeated injections and, through controlled sequential or synchronous release, as profiled in Fig. 5, generates synergistic effects within the RGC-glial functional unit. This approach systematically restores the entire metabolic cascade from energy supply to survival signaling [72].

These microspheres also offer remarkable flexibility in terms of delivery strategies, optimizing convenience and efficacy. For instance, a thermosensitive hydrogel composite of drug-loaded PLGA microspheres prolongs ocular retention after topical application, overcoming the short residence time of traditional eye drops and avoiding invasive injections [73]. Alternatively, microneedle-assisted delivery of brimonidine-loaded PLGA microspheres to the supraciliary space enables targeted and sustained drug release. This approach effectively lowers IOP and minimizes systemic absorption, highlighting the metabolic safety of local precision delivery [74].

## Retinopathy

Retinal diseases, including diabetic retinopathy, retinitis pigmentosa (RP), and proliferative vitreoretinopathy (PVR), are associated with complex metabolic disturbances in the retina. Shared pathologies include persistent oxidative stress, chronic inflammatory imbalance, and impaired neurovascular function because of protein misfolding or ischemia. Current mainstream therapies, such as anti-VEGF injections, require frequent administration, cause nonresponsiveness, and cannot reverse early metabolic damage and neurodegeneration. Thus, there is an urgent need for strategies that can continuously and stably correct retinal metabolic imbalances at their sources.

Metabolically regulatory microspheres meet this requirement by precisely aligning controlled drug pharmacokinetics with pathological metabolic demand. Their core function is to provide sustained and controlled drug release, forming a stable foundation for therapeutic concentrations. For example, puerarin-loaded polylactic acid microspheres leverage polymer degradation to achieve zero-order release kinetics and maintain effective retinal drug levels for weeks, continuously improving microcirculation and suppressing inflammation [75]. Similarly, retinoic-acid-loaded alginate microspheres rely on slow ocular degradation to enable constant release and sustain the suppression of abnormal cellular proliferation in PVR [76].

Besides sustained release, the microsphere carrier design ensures the precision and safety of metabolic regulation. This is critical for agents with narrow therapeutic windows. Porous Se@SiO_2_ nanospheres exemplify this: Their porous silica shell retards selenium dissolution, maintaining intraretinal selenium within a narrow concentration window that fully activates the antioxidant enzyme GPX4 while avoiding toxicity. This carrier-enabled safe supply of selenium is essential for reversing lipid peroxidation [77]. Similarly, tauroursodeoxycholic acid (TUDCA)-loaded PLGA microspheres use a “low-initial-burst-progressive release” profile to address the short half-life of small molecules. The resulting sustained exposure allows TUDCA to function long term as a “chemical chaperone”, continuously correcting the upstream metabolic defect of endoplasmic reticulum protein misfolding in RP [78].

The therapeutic potential of metabolic regulatory microspheres stems from the inherent compatibility between their physicochemical properties as carrier platforms and the metabolic pathologies of retinal diseases. They provide sustained and controllable drug release to overcome pharmacokinetic limitations, ensure precise dosage control for safety, and facilitate codelivery for systematic network regulation. These advanced delivery systems are core components of therapeutic strategies, paving the way for a paradigm that transcends pharmacology to stabilize the retinal metabolic microenvironment.

## Application of Metabolically Active Microspheres in Gynecological Diseases

The maintenance of reproductive endocrine function and tissue homeostasis is governed by the local metabolic microenvironment. Common gynecological conditions, such as premature ovarian insufficiency (POI), ovarian cancer, and endometriosis, share core pathological features, including imbalances in energy metabolism, aberrant proliferation and apoptosis, inflammatory–fibrotic vicious cycles, and dysregulated angiogenesis. Current therapies often fail to precisely reshape the dysfunctional microenvironment, limiting their efficacy in restoring endocrine function and promoting tissue regeneration. Metabolically regulatory microspheres, with their adaptive retention and release profiles aligned with the reproductive tract, offer a novel strategy for correcting critical metabolic abnormalities in both temporal and spatial dimensions.

## Ovarian Diseases

The ovary is central to female reproductive and endocrine functions, and its dysregulation underlies serious pathologies, including POI and ovarian cancer. POI is characterized by a premature decline in the ovarian reserve, massive apoptosis of granulosa cells, follicle depletion, and disrupted hormone synthesis. In contrast, ovarian cancer, particularly advanced chemotherapy-resistant forms, exhibits distinct metabolic reprogramming features, such as dysregulated cholesterol metabolism, the Warburg effect, and hyperactive mitochondrial metabolism, which collectively underlie chemoresistance and metastatic potential [79]. Traditional therapies face significant limitations; hormone replacement therapy for POI alleviates symptoms without restoring the metabolic microenvironment, whereas conventional chemotherapeutics cannot effectively reverse drug-resistant phenotypes sustained by metabolic adaptations.

**Fig. 6.**
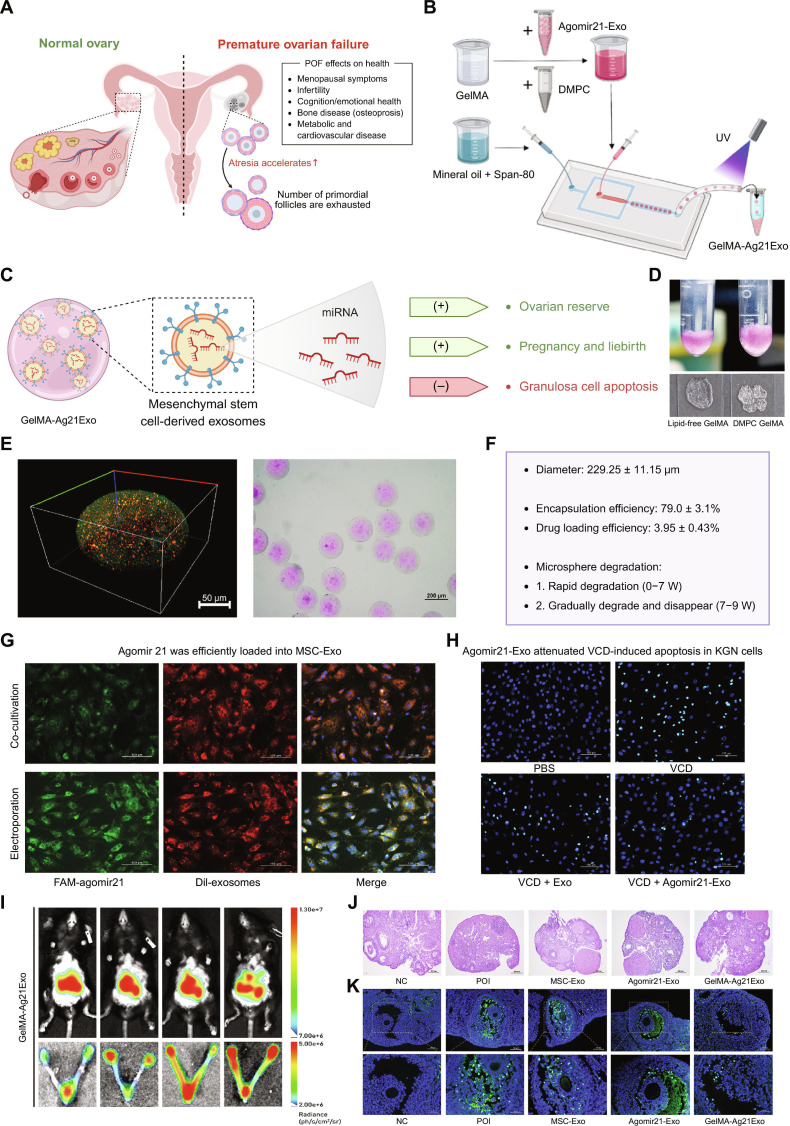
GelMA-Ag21Exo microspheres sustainably released Ag21Exo to ameliorate ovarian function in POI. (A) Premature ovarian failure (POF) is pathologically characterized by accelerated follicular atresia and premature exhaustion of the primordial follicle pool, leading to systemic health issues. (B) Schematic diagram of the fabrication process for GelMA-Ag21Exo using microfluidic techniques. (C) The GelMA-encapsulated exosome system delivers mesenchymal-stem-cell-derived exosomes and miRNAs to ameliorate ovarian reserve and reproductive outcomes while inhibiting granulosa cell apoptosis. (D) Visual appearance of lipid-free GelMA and dimyristoylphosphatidylcholine (DMPC)-GelMA. (E) Confocal microscopy revealed the 3-dimensional structure of GelMA-Ag21Exo, and optical microscopy determined its diameter distribution. (F) The microspheres were characterized by their key physicochemical parameters and a biphasic in vitro degradation profile. (G) Representative micrographs showing differences in the transfection efficiency of cocultivation and electroporation of agomir21. The transfected agomir21 was labeled with fluorescein amidite (FAM) (green), mesenchymal stem cell (MSC)-Exo was labeled with 1,1′dioctadecyl-3,3,3′,3′-tetramethylindocarbocyanineperchlorate (Dil) (red), and cell nuclei were labeled with DAPI (blue). (H) Terminal-deoxynucleotidyl-transferase-mediated deoxyuridine triphosphate nick end labeling (TUNEL) staining showing the number of apoptotic cells in the aforementioned 4 groups. Apoptotic cell nuclei were labeled with deoxyuridine triphosphate (green), and all cell nuclei were labeled with DAPI (blue). (I) Fluorescence imaging trends of GelMA-Ag21Exo in the whole body and reproductive organs of mice. (J) Hematoxylin and eosin staining of ovaries at each stage. NC, normal control; POI, premature ovarian insufficiency. (K) TUNEL staining (green) of the ovaries in each group, representing apoptotic granulosa cells, and DAPI staining (blue), representing all cell nuclei. Licensed under Creative Commons Attribution-NonCommercial-NoDerivatives 4.0 International (CC BY-NC-ND 4.0).

Metabolically regulatory microspheres can address these challenges through targeted interventions. For POI treatment, GelMA-agomir21-Exo (Ag21Exo) microspheres achieved a sustained release of exosomes loaded with an miR-21 agonist, correcting the phosphatase and tensin homolog/Akt pathway imbalance in granulosa cells, restoring glucose metabolism, and inhibiting apoptosis [80]. Figure 6 illustrates the fabrication, key characteristics, and multifaceted therapeutic efficacy of GelMA-Ag21Exo, demonstrating its role in inhibiting granulosa cell apoptosis and restoring the ovarian reserve. Simultaneously, 5β-*O*-angelate-20-deoxyingenol(HEP14)/PLGA microspheres enable continuous HEP14 release to activate the protein kinase C–ERK1/2 pathway in transplanted stem cells, driving ECM remodeling, angiogenesis, and hormonal recovery [81]. For Warburg-effect-dominated tumors, ZrTCP@siFOXK2@CM/Met@GelMA microspheres use a multitarget strategy that combines FOXK2 silencing, AMPK pathway activation, and photothermal therapy to systematically reverse abnormal energy metabolism [33]. Notably, the use of tissue-specific ECM can further enhance the metabolic and regenerative microenvironment. In a recent study, ovarian ECM-derived HMPs (OG-HMPs) have been developed to deliver BMSCs for treating POI. The OG-HMPs not only prolong BMSC retention but also augment their paracrine secretion of proangiogenic and immunomodulatory factors, thereby reprogramming the local metabolic and inflammatory milieu. This approach successfully restores ovarian endocrine function and fertility in a POI mouse model, highlighting the potency of ECM-based metabolic microspheres in restoring tissue-specific metabolic homeostasis [82].

## Endometrial Diseases

Endometrial pathologies, including endometriosis, intrauterine adhesions, and a thin endometrium, share a common foundation for local metabolic reprogramming. This manifests as heightened aerobic glycolysis in immune cells, suppressed proliferative metabolism in repair cells, and aberrant angiogenic metabolism, collectively forming a self-perpetuating “inflammation–metabolism–fibrosis” cycle. Conventional therapies are constrained by poor lesion targeting, short drug half-lives, and misalignment with tissue repair chronobiology, which frequently results in suboptimal outcomes and high recurrence rates.

Microsphere systems enable programmable temporal repair via sophisticated release kinetics. Hierarchically structured dienogest-loaded PLGA microparticles encapsulated in bovine serum albumin hydrogel microparticles achieved near-zero-order dienogest release via a PLGA core–hydrogel shell design, ensuring sustained suppression of ectopic endometrial proliferation [17]. For regenerative applications, 3-dimensional-printed, scaffold-loaded granulocyte colony-stimulating factor microspheres create metabolism-friendly niches that facilitate cellular infiltration and nutrient exchange [83].

Beyond conventional drug delivery, microspheres increasingly function as autonomous metabolic regulators due to their intrinsic physicochemical properties. Cur-Fe_3_O_4_ HMPs exploit photothermal conversion to denature glycolytic enzymes and disrupt mitochondrial function, physically resetting aberrant energy metabolism in ectopic lesions [13]. Platelet-rich plasma-loaded gelatin methacryloyl hydrogel microspheres microspheres serve as metabolic signal concentrators, where the porous architecture protects bioactive factors, and surface modifications directly activate cellular integrin signaling to guide metabolic reprogramming toward regeneration [84].

In summary, metabolically regulated microspheres represent a paradigm shift in gynecological disease management by aligning therapeutic interventions with the inherent metabolic dynamics of the reproductive system. Through the temporal control of drug release, multitarget synergy, and intelligent material design, these systems have transitioned treatment strategies from symptomatic management to fundamental metabolic restoration. This approach effectively addresses the core pathological features of both ovarian and endometrial disorders and establishes a robust foundation for functional recovery in reproductive medicine.

## Challenges and Future Perspectives

Although metabolism-regulating microspheres offer promising therapeutic avenues for correcting disease-specific metabolic dysregulation, their clinical application faces several challenges. The primary hurdles reside not only in achieving precise tissue targeting but also in the intricate interplay between engineered interventions and the host dynamic metabolic network. Key concerns include the risk of off-target metabolic effects, compensatory pathway activation, and significant interpatient metabolic heterogeneity, which collectively complicate the prediction of long-term efficacy and safety of these drugs. Overcoming these barriers necessitates the development of smarter, adaptive microsphere systems capable of intelligent metabolic adaptation, alongside personalized strategies informed by individual metabolic phenotypes.

## Core Challenges

### Tissue-specific delivery efficiency barriers

The therapeutic potential of metabolic regulatory microspheres is constrained by a universal challenge: tissue-specific biological barriers that limit the targeted delivery and sustained efficacy. Although these obstacles vary in form and mechanism, they are ubiquitous across organ systems. They manifest as selective barriers in neural tissues, dynamic fluid microenvironments in reproductive organs, and specialized anatomical protection in the sensory organs. To illustrate this widespread phenomenon, we focused on a representative case of orthopedic applications, where the distinctive structural and mechanical properties of the bone create complex delivery challenges.

Within the orthopedic domain, the delivery and retention of metabolic microspheres face multiple physiological and structural challenges. The densely mineralized bone matrix and compartmentalized vascular network significantly limit microsphere penetration and uniform distribution at target sites [85]. Following systemic or local administration, microspheres frequently encounter restricted access to bone remodeling units owing to architectural constraints coupled with ongoing physiological clearance processes. Furthermore, the dynamic in vivo environment, particularly the composition of the bone marrow and interstitial fluid, often induces accelerated polymer degradation, which markedly differs from observations under standardized in vitro conditions [86]. These interconnected factors collectively undermine the sustained-release profile essential for effective metabolic regulation, including the coordination of osteogenic activity and support of long-term bone remodeling.

Compounding these structural and biological challenges, the dynamic mechanical environment of weight-bearing bones introduces another layer of complexity that is rarely captured by conventional in vitro models. When subjected to cyclic physiological loading during routine activities, such as walking or joint articulation, implanted microspheres experience substantial compressive and shear stresses. These mechanical forces can precipitate premature structural failure, accelerate polymer degradation kinetics, and trigger an unpredictable burst release of bioactive molecules [87]. Such biomechanically driven alterations disrupt the controlled-release profiles of critical metabolic regulators, including BMP-2, parathyroid homone analogs, and antiresorptive agents, ultimately compromising the long-term anabolic or catabolic modulation necessary for addressing osteoporotic bone loss or supporting fracture healing under physiological mechanical demands.

Beyond the skeletal system, metabolic microspheres face equally formidable barriers in ocular and gynecological tissues. The blood–retinal barrier, combined with the viscoelastic properties of the vitreous body, restricts systemic and local microsphere distribution in retinal therapeutics [88]. Parallel challenges have emerged in ovarian and endometrial applications, where selective vascularization patterns, mucosal layers, and dynamic follicular fluid environments collectively impede the access and retention of targeted microspheres. These organ-specific characteristics systematically undermine spatial control and sustained metabolic interventions at the intended pathological sites.

These multifaceted delivery challenges, spanning the structural, biological, and mechanical barriers in the bone, along with the selective permeability and complex fluid dynamics characterizing the retinal and reproductive tissues, collectively constrain the translational potential of metabolic microspheres. This limitation restricts their capacity to provide durable and localized metabolic regulation across a spectrum of chronic diseases that affect these diverse organ systems.

### Individual variability

The therapeutic efficacy of metabolic regulatory microspheres is limited by physical and biological barriers and is profoundly influenced by another critical factor: substantial heterogeneity in metabolic profiles among individual patients and across different disease stages. This variability poses a fundamental challenge in developing universally effective microsphere formulations.

In OA, cartilage explants from affected patients exhibit marked differences in the proteolytic pathways that mediate proteoglycan degradation, even under identical culture conditions. The release of sulfated glycosaminoglycans (sGAGs) varied significantly between patient subgroups; one group showed MMP-3-dependent sGAG loss and the other showed a disintegrin and metalloproteinase with thrombospondin motifs 5 (ADAMTS-5)-dependent loss, whereas the anti-inflammatory cytokine interleukin-37 (IL-37) selectively reduced sGAG release only in the MMP-3-dependent subgroup. This suggests that cartilage degradation is not uniformly regulated across patients with OA and that key proteases, such as MMP-3 and ADAMTS-5, contribute differently depending on the metabolic profile of the individual. Such metabolic heterogeneity implies that microspheres designed to modulate chondrocyte activity, for instance, by supporting anabolic functions or suppressing catabolic pathways, must be tailored to match the specific metabolic state and biosynthetic capacity of the target tissue [89,90]. A standardized, “one-size-fits-all” microsphere system with fixed drug loading and release kinetics is likely to fail to account for these individual differences, leading to inconsistent therapeutic outcomes and limited clinical translation.

Similar challenges are observed in oncology and metabolic diseases, where tumor subtypes or disease stages exhibit distinct metabolic dependencies, such as variations in glycolytic flux or mitochondrial respiration. These differences determine not only the choice of therapeutic agent but also the required release profile and the local dosage. Microsphere-based interventions are ineffective in a significant proportion of the target population without prior stratification of patients based on metabolic signatures or tissue biomarkers.

### Complexity and long-term safety of metabolic networks

Cellular metabolism operates as a highly interconnected and adaptive network, where targeted intervention in one pathway frequently triggers compensatory activation of alternative routes or systemic remodeling. This inherent redundancy and plasticity pose dual challenges: not only may monotherapeutic strategies fail to achieve sustained efficacy, but they may also induce unpredictable metabolic shifts that compromise long-term safety.

For instance, in OA, suppression of glycolysis in chondrocytes, intended to reduce inflammatory metabolite accumulation, can inadvertently disrupt mitochondrial respiration and TCA cycle flux, impair matrix synthesis, and accelerate cellular senescence [91]. In tumor metabolism, inhibiting glutaminase may suppress tumor growth and ultimately promote resistance by enriching the populations reliant on fatty acid oxidation or other amino acids [92]. These adaptive responses highlight that microspheres delivering a single metabolic modulator are unlikely to durably control pathological processes and may even drive disease evolution or alter the phenotypic presentation.

However, the long-term effects of these metabolic interventions remain unclear. Sustained modulation of core pathways, such as glycolysis or lipid metabolism, in bone microenvironments to promote osteogenesis could perturb the delicate balance of bone remodeling or indirectly affect hematopoiesis in the adjacent marrow [92]. In retinal disorders, continuous alterations in photoreceptor energy metabolism induce metabolic exhaustion in neighboring cells [93]. Besides these biological consequences, the materials and degradation products of metabolic microspheres may exhibit chronic immunogenicity or accumulate in off-target tissues, raising additional concerns.

Therefore, a comprehensive understanding of network-level metabolic adaptations, combined with a rigorous evaluation of long-term biocompatibility and systemic effects, is essential for designing effective and safe microsphere-based therapies. The translation of such approaches will depend not only on the spatial and temporal control of drug release but also on anticipating dynamic host responses and minimizing unintended metabolic disturbances in the host.

## Emerging Directions

### Personalized metabolic microspheres

The development of personalized metabolic microspheres represents a paradigm shift from standardized drug delivery to precision therapy tailored to the pathophysiology of individual patients. This approach directly addresses the fundamental challenge of metabolic heterogeneity, in which patients with nominally similar diagnoses exhibit markedly different drug responses owing to variations in the tissue microenvironment, metabolic activity, and clearance mechanisms.

The FX006 extended-release microsphere system for intra-articular triamcinolone acetonide delivery provides a compelling case study of how tissue fluid analysis can guide formulation optimization [94]. Clinical evaluations involving systematic synovial fluid sampling have revealed critical interpatient variations in drug pharmacokinetics. While the microsphere formulation prolonged synovial residency compared to conventional suspensions, the actual drug concentrations exhibited substantial variability among patients at each time point. Some patients maintained therapeutic concentrations beyond 12 weeks, whereas others showed a rapid decline to subtherapeutic levels by week 6. This heterogeneity directly reflects the differences in individual joint environments, including variations in synovial inflammation, enzymatic activity, and fluid turnover, which conventional formulation approaches have ignored.

The critical insight from this study is that effective personalization requires an understanding of drug concentrations and the underlying metabolic and inflammatory contexts that determine drug stability and activity. However, the current limitation of this approach lies in its reactive nature: Microsphere parameters are fixed during manufacturing, whereas synovial fluid analysis occurs postadministration, providing insights for future formulations rather than enabling real-time adjustment. Next-generation personalized microspheres leverage pretreatment tissue fluid profiling to customize formulation characteristics [95]. The analysis of synovial fluid, vitreous humor, or cerebrospinal fluid can quantify patient-specific factors, such as protease concentrations, pH variations, oxidative stress markers, and metabolic waste products that influence drug stability and release kinetics. This diagnostic information directly guides the selection of polymer composition, degradation rate, and drug loading in microspheres tailored to an individual’s tissue microenvironment.

The goal is to create adaptive microsphere systems that can respond to changing metabolic conditions within target tissues [96]. Future platforms should incorporate environmentally responsive polymers that modulate drug release in response to fluctuating inflammatory markers and metabolic stress indicators. Combined with point-of-care diagnostic systems that rapidly analyze patient tissue fluids before treatment, this approach enables personalized metabolic interventions, ensuring that optimal drug exposure matches each patient’s unique and dynamic disease states.

This evolution from population-based to patient-specific delivery strategies promises to overcome the fundamental limitations of current microsphere systems, transforming the management of complex metabolic disorders by precisely targeting individual pathological mechanisms.

### AI-driven design

The development of personalized metabolic microspheres has been accelerated by AI and machine learning (ML), which provide diagnostic insights and therapeutic customization [97]. A compelling illustration comes from a recent study on postmenopausal OP, in which ML algorithms have been used to analyze serum metabolic fingerprints obtained via hierarchical porous microsphere-assisted laser desorption/ionization mass spectrometry [98]. By processing 673 metabolic features from 200 individuals, 6 ML models, including orthogonal partial least-squares discriminant analysis, *k*-nearest neighbor, and random forest, distinguished between healthy controls, osteopenia, and different stages of OP, with area under the curve values of >0.98. The models identified 7 key mass/charge ratio features associated with disease progression, including fumarate, citrulline, and asymmetric dimethylarginine, which are involved in arginine biosynthesis and the TCA cycle and regulate bone metabolism. This finding demonstrates the capacity of ML not only to classify complex metabolic phenotypes but also to identify critical biomarkers that can guide the composition and release profiles of therapeutic microspheres.

Such an AI-driven approach has numerous advantages. ML models can integrate multiomics data—metabolomic, proteomic, and genomic—to identify nonobvious correlations between microsphere physicochemical properties (e.g., polymer composition, porosity, and surface charge) and their in vivo performance [99–101]. This enables the in silico optimization of microsphere formulations, significantly shortening the development cycles that traditionally rely on trial-and-error experimentation. Furthermore, AI facilitates the design of adaptive microsphere systems capable of modulating drug release in response to dynamically changing local metabolite levels, enabling real-time metabolic interventions tailored to a patient’s disease activity [102].

The integration of AI with real-time biosensing and closed-loop feedback systems is thus crucial. Future intelligent microspheres could be designed to continuously monitor local metabolic markers and adapt their release kinetics using built-in responsive materials. As multimodal data become more accessible and ML models become more interpretable and robust, AI-driven design is poised to transform metabolic microspheres from static delivery vehicles into dynamic patient-specific therapeutic systems that maintain metabolic homeostasis in an individualized and adaptive manner.

However, the development of such adaptive systems first hinges on the ability to accurately stratify patients based on their distinct metabolic pathologies. Translating this AI-driven paradigm into practice requires prioritizing the most actionable biomarkers for initial patient stratification. For diseases interfacing with body fluids, such as OA within the joint cavity, synovial fluid metabolomic profiling stands out as a critical and feasible primary data source. As evidenced by the interpatient variability in synovial fluid drug pharmacokinetics (e.g., with FX006 microspheres), this microenvironment directly mirrors the local pathological metabolic state. Systematic, longitudinal metabolomic analysis of synovial fluid can quantify a spectrum of disease-relevant metabolites, including glycolytic intermediates, TCA cycle components, oxidative stress markers, and inflammatory lipid mediators. Correlating these high-dimensional metabolomic signatures with clinical outcomes will generate the essential dataset needed to train robust ML models for patient stratification. This data-driven approach can then inform the selection of key microsphere parameters, such as antioxidant potency, anti-inflammatory dose, or specific anabolic cues to tailor the “metabolic command” to each patient’s synovial environment. Establishing such a fluid-biomarker-guided framework represents a concrete near-term research priority to bridge computational design and clinically personalized microsphere therapies.

### Organ-on-a-chip and organoids as predictive validation platforms

Organ-on-a-chip (OoC) and organoid technologies collectively represent a transformative approach for addressing the critical limitations of conventional in vitro models for evaluating metabolic microspheres, particularly their inability to recapitulate tissue-specific biological barriers, dynamic mechanical environments, and native tissue architecture [103]. By simulating human physiology across different dimensions, these 2 platforms establish a biologically relevant bridge between simplified cell cultures and complex in vivo systems, enabling more accurate prediction of microsphere behavior and metabolic interventions.

OoC technology offers unique advantages in simulating biomechanical cues, such as fluid shear stress and cyclic mechanical strain, making it particularly suitable for mimicking physiological processes, such as cyclic loading within the joint cavity or hemodynamics in the vasculature. A prominent example of this strategic application is found in regulatory science, where the US Food and Drug Administration’s new approach methodology roadmap advocates the use of human-based microphysiological systems. For instance, in the context of metabolic microspheres designed for musculoskeletal disorders, a bone-on-a-chip model can not only emulate the multicellular architecture involving osteoblasts, osteoclasts, and chondrocytes but also incorporate dynamic mechanical loading and inflammatory cytokine gradients that closely mimic the pathophysiological joint environment. This system enables real-time monitoring of microsphere degradation kinetics and drug release profiles under conditions that replicate local mechanical and metabolic stress, overcoming the key limitations of static in vitro cultures [104]. Similarly, a skeletal muscle-on-a-chip platform integrates vascular endothelial components and neuromuscular junctions within a highly organized contractile muscle tissue model, allowing the evaluation of microsphere localization, myofiber-specific metabolic modulation, and the influence of muscle activity on release kinetics in a human-relevant setting [105].

Beyond serving as foundational tissue models, OoC platforms are particularly powerful for validating microsphere performance against the complex, multifactorial cues of specific diseases. For example, in OA research, these systems effectively model key disease drivers. “Cartilage-on-a-chip” models have applied hyperphysiological compression (e.g., 30% strain at 1 Hz) to mimic the mechanical overload central to posttraumatic OA. In parallel, other models establish gradients of proinflammatory cytokines (e.g., tumor necrosis factor-α and IL-1β) to simulate the chronic inflammatory milieu of synovial fluid [106]. Building on these established paradigms, testing metabolism-regulating HMPs within a calibrated OA-on-a-chip system provides direct insights for design optimization. Subjecting microspheres to defined cyclic mechanical strain reveals whether joint-like loading accelerates polymer fatigue or induces premature drug release, thereby guiding the enhancement of cross-linking density and material toughness to ensure mechanical robustness. Simultaneously, exposing them to a cytokine gradient within this dynamic environment tests the fidelity of their stimulus-responsive (e.g., ROS-sensitive) release mechanisms, enabling the fine tuning of trigger thresholds and release kinetics against a pathologically relevant background.

Organoids, formed via the self-organization of stem cells into 3-dimensional miniaturized organs, exhibit great potential for recapitulating the cellular diversity, native microarchitecture, and endogenous metabolic functions of specific tissues and organs. For example, articular cartilage organoids derived from patient-specific induced pluripotent stem cells spontaneously develop characteristic lacunar structures and tissue-specific extracellular matrices, providing an ideal model for investigating the metabolic regulatory functions of microspheres, such as correcting glycolytic flux and inhibiting abnormal hypertrophy in an authentic cartilage-like environment [107]. Similarly, skeletal muscle organoids mimic the key stages of in vivo muscle development, forming contractile myotube bundles that are highly suitable for evaluating the long-term efficacy of metabolic microspheres in promoting myofiber regeneration and modulating lipid metabolism [108].

The integration of OoC and organoid technologies by embedding organoids into microfluidic chips with perfusable flow and mechanical stimulation constitutes a cutting-edge validation strategy. These OoC systems combine the biological complexity of organoids with the physiological dynamism of OoC platforms, resulting in an unprecedented and highly biomimetic human physiology simulator [109,110]. When applied to metabolic microspheres, such integrated platforms can reveal patient-specific issues that are undetectable in static cultures or animal models, such as accelerated polymer hydrolysis in inflammatory microenvironments and insufficient drug release under mechanical strain, thus paving the way for personalized microsphere design in the preclinical stage.

The deep integration of OoC, organoids, AI-driven modeling, and high-content metabolomics opens new avenues for personalized metabolic microsphere therapies. These systems will not only help overcome the historical disconnect between in vitro performance and in vivo efficacy but also establish a robust human biology-centric framework for designing metabolic microspheres that are both safe and effective across diverse patient populations.

## Conclusion

Metabolic regulatory microspheres represent a paradigm shift from conventional drug delivery systems to active platforms capable of spatiotemporally reprogramming pathological tissue microenvironments. This review describes the engineering of chemical and physical properties, including surface functionalization, stimuli-responsive release, and biomechanical cues, enabling precise correction of dysregulated metabolic pathways across the musculoskeletal, reproductive, and visual systems. These systems demonstrate significant potential for restoring metabolic homeostasis in a localized and sustained manner by targeting core mechanisms, such as glycolytic flux, mitochondrial dysfunction, and oxidative stress. Despite promising advances, critical challenges remain, including overcoming tissue-specific delivery barriers, accounting for interindividual metabolic heterogeneity, and ensuring long-term safety within dynamic metabolic networks. Future progress will depend on the integration of personalized diagnostic profiling, AI-driven design, and human-relevant validation platforms such as OoC technologies. Through continued interdisciplinary collaboration and a deeper understanding of host–material metabolism interactions, metabolic microspheres have the potential to establish a new therapeutic modality for the precise treatment of a broad spectrum of chronic diseases driven by metabolic dysregulation.
